# Network Consistency Projection for Human miRNA-Disease Associations Inference

**DOI:** 10.1038/srep36054

**Published:** 2016-10-25

**Authors:** Changlong Gu, Bo Liao, Xiaoying Li, Keqin Li

**Affiliations:** 1 College of Information Science and Engineering, Hunan University, Changsha, Hunan 410082, China; 2 Department of Computer Science, State University of New York, New Paltz, New York 12561, USA

## Abstract

Prediction and confirmation of the presence of disease-related miRNAs is beneficial to understand disease mechanisms at the miRNA level. However, the use of experimental verification to identify disease-related miRNAs is expensive and time-consuming. Effective computational approaches used to predict miRNA-disease associations are highly specific. In this study, we develop the Network Consistency Projection for miRNA-Disease Associations (NCPMDA) method to reveal the potential associations between miRNAs and diseases. NCPMDA is a non-parametric universal network-based method that can simultaneously predict miRNA-disease associations in all diseases but does not require negative samples. NCPMDA can also confirm the presence of miRNAs in isolated diseases (diseases without any known miRNA association). Leave-one-out cross validation and case studies have shown that the predictive performance of NCPMDA is superior over that of previous method.

MicroRNAs (miRNAs) are a class of small non-coding RNAs which are about 20 to 25 nucleotides long. miRNAs are important regulatory RNAs, which mainly function in repressing gene expression at the post-transcriptional level by binding to the 3′-UTR of target mRNAs through base pairing^1^,^2^. However, some researchers found out that miRNAs may also positively regulate target mRNAs^3^. Substantial evidence indicated that miRNA dysregulation is related to a number of human diseases, such as cancer^4^,^5^. miRNAs affect human diseases by the interaction with various factors, such as miRNA-mRNA interactions^6^, miRNA-protein interactions^7^, miRNA-lncRNA (long non-coding RNA) interactions^8^,^9^, miRNA-environmental factors interactions^10^,^11^, and so on. More miRNA-disease associations have been reported in the last few years. By collecting data from experiments supporting human miRNAs and disease associations from published studies, Li *et al*.^12^ and Jiang *et al*.^13^ constructed two comprehensive databases, namely, Human miRNA-associated Disease Database (HMDD) and miR2Disease, respectively. Yang *et al*.^14^ presented a database of differentially expressed miRNAs in human cancers (dbDEMC) to explore the expression of aberrant miRNAs in different cancer conditions. Therefore, identifying disease-related miRNAs (disease miRNAs) will not only help in the investigation of the pathogenesis of diseases at the molecular level^15^ but will also facilitate the diagnosis, treatment, and prevention of diseases.

In recent decades, the prediction and ranking of disease-related miRNAs have received much attention^16^,^17^,^18^,^19^. Experimental verification method for the identification of disease-associated miRNAs is expensive and time-consuming^20^,^21^. However, given the large number of studies available, immense biological data about miRNAs have been generated, providing a strong basis for the development of powerful computational methods to predict novel human miRNA-disease associations on a large scale^22^. Such computational methods mainly aim to predict interactions between diseases and miRNAs^23^. The key problem for miRNA-disease association inference is similarity computation, some studies developed computation methods to measure miRNA similarity and disease similarity, Zou *et al*.^24^ reviewed the main similarity computation methods and the future works of them. Among these computational methods, machine-learning-based methods and network-based methods are the main representatives.

Machine-learning-based models and algorithms have been used to solve the problem in practice, and it is beneficial to improve the classification accuracy and prediction performance^25^,^26^. Several studies have proposed machine-learning-based methods to predict novel miRNA-disease associations. Ala Qabaja *et al*.^27^ used the Lasso regression model of protein interaction to find associations between miRNAs and diseases. Jiang *et al*.^28^ proposed a NaïveBayes model to prioritize disease-related miRNAs through genomic data integration. To distinguish positive miRNA-disease associations from negative ones, Jiang *et al*.^21^ proposed a support vector machine (SVM) classification method. Xu *et al*.^29^ recommended a prediction method by conducting functional enhancement of miRNA-target dysregulated network. Zeng *et al*.^30^ proposed two multipath methods to predict disease related genes based on gene-disease heterogeneous network and then applied to predict miRNA-disease associations^31^, and achieved good results. Unfortunately, such machine-learning-based approaches face a common limitation; the negative training samples consisting of non-association between miRNAs and diseases do not demonstrate sufficient statistical confidence because an association was not observed in a biological experiment that cannot draw a conclusion indicating no association between them. Considering that the negative training samples are difficult to obtain, Chen *et al*.^32^ developed the Regularized Least Squares for miRNA-Disease Associations (RLSMDA) to prioritize the discovery of potential miRNA-disease associations without utilizing negative samples. RLSMDA is a semi-supervised classification algorithm that can predict associations for isolated diseases. To predict different types of miRNA-disease associations, Chen *et al*.^33^ developed the model of Restricted Boltzmann Machine for Multiple types of MiRNA-Disease Association prediction (RBMMMDA). RBMMMDA can effectively predict different types of miRNA-disease associations.

Recently, more researchers started using the network-based approaches to predict relationships between miRNAs and diseases. These methods sort the results of the prediction, and recommend some of the previous entries to biologists for further validation. As a result, these methods can be regarded as a recommender system, which has a wide range of applications in many other fields such as movies, news and social tags^34^. The network-based methods to predict miRNA-disease associations are based on the common assumption that miRNAs with similar functions are normally associated with phenotypically similar diseases and vice versa^35^,^36^. On the basis of this assumption, Jiang *et al*.^20^ constructed a miRNA functional network and predicted potential miRNA-disease associations by phenotype similarity through hyper-geometric distribution of miRNA-target genes. Given that miRNA-target genes demonstrate a high probability of yielding false-positive results, the same probability of bearing false-positive results is highly possible in predicting miRNA-disease associations. By using data on miRNA-disease associations and the directed acyclic graph (DAG) of disease annotation, Wang *et al*.^37^ presented a method to calculate the miRNA functional similarity (referred to as MISIM) and constructed a network of miRNA functional similarities. However, considering the incomplete description of diseases, the miRNA similarities calculated by target-disease annotation appear too biased. Considering miRNA functional similarity and the known miRNA-disease associations simultaneously, Chen *et al*.^38^ developed the model of within and between score for miRNA-disease association prediction (WBSMDA), WBSMDA could predicted associations with disease without any known related miRNA. Liu *et al*.^39^ measured disease similarity and miRNA similarity by integrating multiple data sources and constructed a heterogeneous network using the known miRNA- disease associations. They extended random walk with restart to predict miRNA-disease associations in the heterogeneous network. The cross validation and case studies show a good performance for predicting potential miRNA-disease associations. Chen *et al*.^22^ adopted a universal network similarity measure to construct a miRNA–miRNA functional network and then proposed the Random Walk with Restart for miRNA-Disease Association (RWRMDA) to predict potential miRNA-disease associations. Chen *et al*.^16^ implemented a random walk on the Online Mendelian Inheritance in Man (OMIM) database^40^ disease similarity network to infer potential associations between diseases and miRNAs. Unfortunately, when a random walk is applied to a particular disease, the miRNA-related information is disregarded. In addition, RWRMDA does not recognize the miRNA family or cluster information and is incapable of predicting novel miRNAs for diseases without any known related miRNAs (isolated diseases). Xuan *et al*.^41^ presented an algorithm (called HDMP) to predict miRNA-disease associations. HDMP measures miRNA–miRNA functional similarity based on the k of highly similar miRNAs and the distribution information of miRNA verified through experiments of these miRNA groups. Recently, Chen *et al*.^42^ presented the network-consistency-based inference (NetCBI) method, which was based on global network measure, to predict potential miRNA-disease associations. NetCBI integrated miRNA similarities, disease similarities, and known miRNA-disease associations to construct a globally associated network for predicting miRNA-disease associations. NetCBI can also predict miRNA-disease associations in isolated diseases, but the performance of cross validation is not as good as that of RWRMDA.

According to previous narratives, the existing computation methods for predicting miRNA-disease associations are restricted by the following limitations. First, some machine-learning-based methods require negative samples that are difficult to obtain. Second, some approaches are unable to predict isolated disease-related miRNAs. Third, some approaches do not recognize the positive influence of miRNA family or cluster information. Finally, although some methods such as NetCBI can predict isolated diseases, their cross-validation performance is poor.

To solve these complications, we propose a method called Network Consistency Projection for miRNA-Disease Associations (NCPMDA) in this paper. NCPMDA calculates the score of each miRNA-disease pair by integrating the miRNA functional similarity network, the disease semantic similarity network, the known miRNA-disease associations, and the miRNA family information to discover the potential associations. NCPMDA shows a clear advantage over other methods, which involve various features, such as leave-one-out cross validation, case studies, global prediction for all diseases, and prediction of novel miRNAs for isolated diseases.

The main contributions of the paper are summarized as follows.

1. NCPMDA is a simple and effective method to predict the associations between miRNAs and diseases by integrating various molecular data.
2. NCPMDA is a non-parametric method.

## Results

### Leave-one-out cross validation of NCPMDA

In this study, leave-one-out cross validation (LOOCV) was implemented on known and experimentally verified miRNA-disease associations to evaluate the predictive performance of NCPMDA. NCPMDA was tested using the benchmark dataset to assess its power and infer potential miRNA-disease associations. To deduce a miRNA-disease association, the known association was left out, and the remaining associations were used as a training set to recover predictive score of the association. Moreover, we used the area under the receiver operating characteristic (ROC) curve (AUC) to evaluate the performance of the methods. The closer AUC is to 1, the better the predictive performance is. ROC curve plots test sensitivity or true-positive rate (TPR) versus 1-specificity or false-positive rate (FPR) at different thresholds. The sensitivity refers to the percentage of the test miRNAs with ranking above a given threshold, specifically the ratio of the successfully predicted miRNA-disease associations to the total experimentally verified miRNA-disease associations. Specificity refers to the percentage of associations below the threshold. However, considering the limited number of known and experimentally verified miRNA-disease associations, using only AUC to evaluate the performance of predictive method was too arbitrary; thus, we also used precision-recall (PR) curve and the area under PR curve (AUPR) to complement the performance evaluation. Generally, if the ROC curve and the PR curve show similar variation and AUPR more close to 1, the prediction performance is better. The PR curve plots precision versus recall at different thresholds. In PR-curve plots, the precision refers to the ratio of correctly predicted associations to all associations with scores higher than the given threshold; by contrast, the recall refers to the ratio of correctly predicted associations to all known miRNA-disease associations.

**Figure 1:**
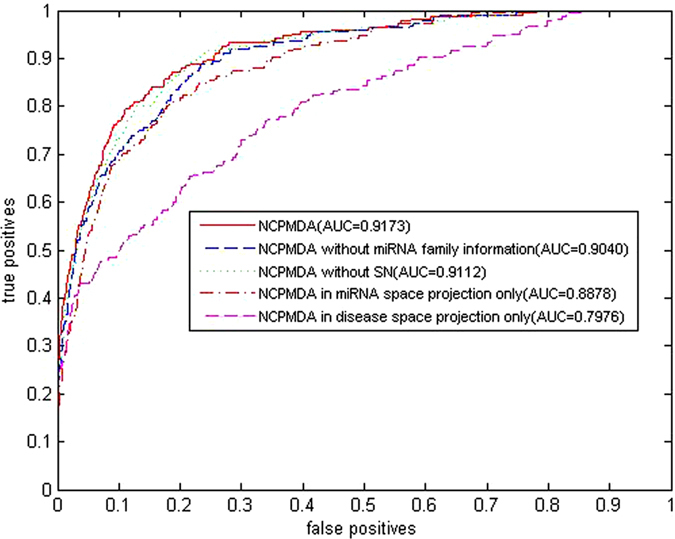
ROC curves and AUC values of NCPMDA based on LOOCV in different situations. (1) NCPMDA with all information (NCPMDA), (2) NCPMDA without miRNA family information, (3) NCPMDA without SN, (4) NCPMDA in miRNA space projection only, (5) NCPMDA in disease space projection only. SN is the similarity of the known miRNA-disease association network.

**Figure 2:**
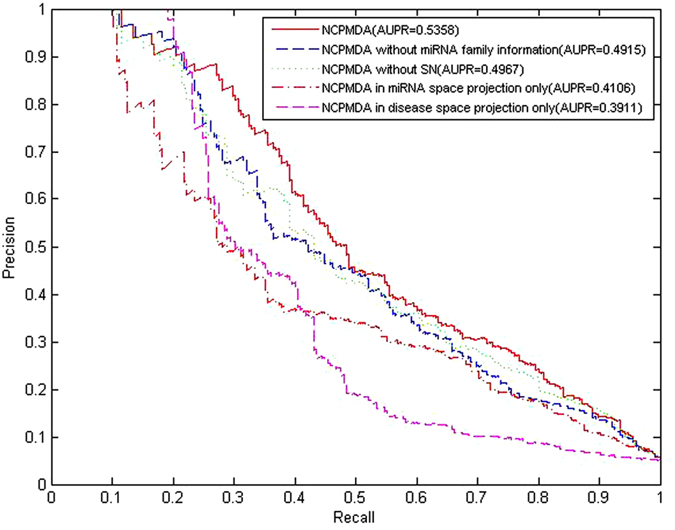
PR curves and AUC values of NCPMDA based on LOOCV in different situations. (1) NCPMDA with all information (NCPMDA), (2) NCPMDA without miRNA family information, (3) NCPMDA without SN, (4) NCPMDA in miRNA space projection only, (5) NCPMDA in disease space projection only. SN is the similarity of the known miRNA-disease association network.

On the basis of miRNA functional similarities and disease semantic similarities, NCPMDA integrates the similarity of known miRNA-disease association network (SN) and miRNA family information to construct a global miRNA-disease network for predicting miRNA-disease associations. We tested the predictive performance of NCPMDA considering the following aspects: (1) NCPMDA with all information (NCPMDA); (2) NCPMDA without miRNA family information; (3) NCPMDA without SN; (4) NCPMDA in miRNA space projection only; (5) NCPMDA in disease space projection only. The ROC curves of the above mentioned features are plotted in Fig. 1, and the PR curves are represented in Fig. 2.

Obviously, NCPMDA exhibits a commendable predictive performance with an AUC value of 0.9173. The miRNA family information is advantageous in improving the predictive performance of NCPMDA. The SN increases the AUC value from 0.9112 to 0.9173 and obviously improves AUPR by increasing it from 0.4967 to 0.5358. In a single-space projection (miRNA space projection or disease space projection) test, NCPMDA also performs well with AUC values of 0.8878 in miRNA space projection and 0.7976 in disease space projection. If SN is removed from NCPMDA, the predictive performance is reduced. Therefore, the predictive performance of miRNA-disease associations is practical to improve by integrating the SN and miRNA family information in the method.

### Comparison with other methods

To the best of our knowledge, HDMP^41^, RLSMDA^32^, NetCBI^42^, and the global network algorithm developed by Shi *et al*.^43^ are state-of-the-art computational methods for predicting miRNA-disease associations. However, HDMP does not work for diseases without known related miRNAs; thus, this algorithm cannot be used for comparisons. With regard to the method developed by Shi *et al*. in constructing a global network by integrating disease gene associations, miRNA-target interactions, and protein interactions to predict miRNA-disease associations, the datasets vary significantly from the ones used in our method. Furthermore, they did not use known miRNA-disease associations; thus, the performances of this method and NCPMDA cannot be fairly and reasonably compared. NCPMDA, RLSMDA, and NetCBI, which were developed using similar datasets, can be used to predict novel miRNA-disease associations for diseases with no known miRNAs. On the basis of the above analyses, we compared the performance of NCPMDA with those of RLSMDA and NetCBI.

**Figure 3:**
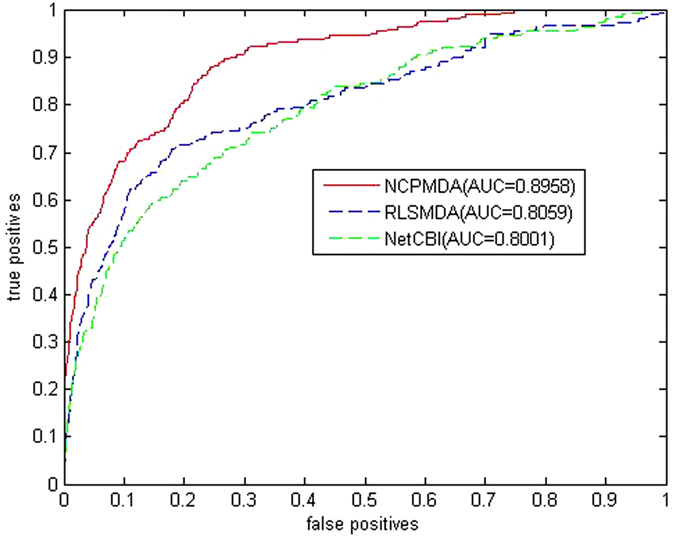
ROC curves and AUC values of NCPMDA, RLSMDA and NetCBI. Without considering the similarity of the known miRNA-disease association network and miRNA family information, NCPMDA performs better than that of RLSMDA and NetCBI in LOOCV.

NCPMDA, RLSMDA, and NetCBI were tested on the benchmark dataset to assess their performance in deducing the potential miRNA-disease associations. We implemented a LOOCV for each method. The optimal parameters are selected for RLSMDA and NetCBI as described in the literature. Given that RLSMDA and NetCBI do not employ the similarity of known miRNA-disease associations and miRNA family information, the three methods were assessed using only miRNA functional similarities and disease semantic similarities to predict miRNA-disease associations. Figure 3 shows the ROC curves and the AUC values to predict the miRNA-disease associations by using the three methods. Without considering the similarity of the known miRNA-disease association network and miRNA family information (Fig. 3), the AUC value of NCPMDA is 0.8958, and the AUC values of RLSMDA and NetCBI are 0.8059 and 0.8001, respectively. Evidently, NCPMDA performs better than that of RLSMDA and NDBM in LOOCV.

To avoid data dependence, the generalization abilities and strength of the algorithms were further verified on the predictive dataset based on LOOCV. The AUC value of NCPMDA disregarding the similarity of the known miRNA-disease association network and the miRNA family information is 0.9605, and the AUC values of RLSMDA and NetCBI are 0.9511 and 0.9560, respectively. The AUC values of the three methods on the predictive dataset are higher than those on the benchmark dataset because miRNA-disease associations differ significantly. In the benchmark dataset, each miRNA is associated with 2.27 disease phenotypes, and each disease phenotype is associated with 4.41 miRNAs on the average. However, in the predictive dataset, the values are 5.147 and 10.18, respectively. Given the increase in the number of associations that the known miRNA-disease associations make which are closer to the real network, the predictive performance is better. For the same reason, the predictive performance of NCPMDA disregarding the similarity of the known miRNA-disease association network consistency and miRNA family information was excellent. NCPMDA also performs slightly better than the two other methods on the predictive dataset. Therefore, our method demonstrates strong data generalization ability. Our algorithm also shows an obvious advantage when the known and experimentally verified miRNA-disease associations are very few.

### Comprehensive prediction of unknown associations

The predictive performance of our method is thoroughly discussed in the first section of this paper. In this section, we utilized NCPMDA to predict unknown miRNA-disease associations, including all possible miRNA-disease pairs from the predictive dataset. The reason for not using the benchmark dataset to do the prediction is that the benchmark dataset contains fewer miRNA numbers, many of these miRNAs associated with disease has been confirmed by the updated databases, which will lead to a very high accuracy of prediction. First, the projection score of each miRNA-disease pair was calculated using all known and verified miRNA-disease associations, similarity information, and miRNA family information. Second, the unknown associations were ranked according to the projection scores. Finally, the top 40 associations were manually verified through three online databases: HMDD^12^, miR2Disease^13^, and dbDEMC^14^ (The database is being updated, and the experimentally validated miRNA-disease associations were gained from the author.). The predictive results and verified evidences are presented in Table 1. Among the top 40 predictive associations, only four have not been confirmed in the aforementioned three databases and the top 10 were all confirmed.

### Case studies of Breast cancer and Hepatocellular cancer

Increasing evidence indicates that miRNAs play critical roles in the development of breast cancer and hepatocellular carcinoma (HCC). Case studies were analyzed to further evaluate the ability of NCPMDA to predict miRNA-disease associations. Similarly, all known associations were used as training set, and the unknown associations were assigned as the testing set. The predictive results were manually verified by three online databases. The top 40 potential breast cancer and HCC-related miRNAs predicted by NCPMDA are listed in (Supplementary Informations S1 and S2), respectively.

Breast cancer is one of the most common form of cancer among women. In the predictive dataset, 78 miRNAs are associated with breast cancer. The potential breast cancer-related miRNAs were predicted by NCPMDA based on the 78 known associations. Among the top 40 predicted miRNAs, 38 miRNAs were confirmed by the three aforementioned databases; and only hsa-mir-30e and hsa-mir-142 were not confirmed. However, Lin *et al*.^44^ demonstrated that has-miR-30e were down regulated in both plasma and breast cancer tissues, as described in the literature^45^, which provided information that has-mir-142 inhibited breast cancer cell invasiveness. Given that this evidence came in after the last updates for the three databases, they were not incorporated in the databases in time for the study. The evidence found in the literature further demonstrated the reliability of NCPMDA in predicting new disease-related miRNAs.

HCC is the most common type of liver cancer. HCC most commonly occurs in countries where hepatitis B infections are common. A total of 34 HCC-related miRNAs are found in the predictive dataset. Among the top 40 HCC-related miRNAs predicted by NCPMDA, 37 have been confirmed by HMDD, mir2disease, and dbDEMC. Further evidences are found to support our prediction results. Using quantitative methylation analysis and real-time PCR, Tang *et al*.^46^ defined has-miR-429 as a key inducer for HCC pathogenesis and metastasis with potential utility for tumor intervention. Based on gene knockdown experiment, Jung *et al*.^47^ summarized that Gα12gep oncogene inhibits FOXO1 in HCC is caused by miR-135b and miR-194 dysregulation. Using methylation-specific PCR, Xie *et al*.^48^ suggest that DNA methylation may be involved in the inactivation of miR-34 b in HCC.

### Application of NCPMDA to predict isolated diseases

An isolated disease refers to a disease without any known associated miRNA. To demonstrate the predictive ability of NCPDMA on isolated diseases, we removed the known and verified miRNA-disease associations related to predictive diseases. This operation ensured that we only used similarity information and known miRNA-disease associations of the other diseases to predict disease-related miRNAs. We conducted case studies on breast cancer and HCC, and the results are presented in (Supplementary Informations S3 and S4), respectively. For breast cancer, we removed 78 known miRNA-breast cancer associations to predict the unknown associations by NCPMDA. Out of the top 40 predicted miRNAs, 37 have been confirmed based on the updated HMDD, mir2disease, and dbDEMC databases. For HCC, 34 known miRNA-HCC associations were removed, and 36 of the top 40 predicted miRNAs were confirmed. Moreover, the top 20 predictions for breast cancer and HCC were all confirmed. Therefore, NCPMDA performs well in the prediction of isolated disease.

## Discussions

Accumulative evidence indicated that miRNAs play important roles in the occurrence and development of diseases. Identification of disease-related miRNAs helps in understanding the mechanism of diseases. Effective computational methods for identifying miRNA-disease associations can provide support for experimental studies on miRNAs.

In this study, we presented a network-based approach (NCPMDA) to predict miRNA-disease associations. On the basis of miRNA similarities and disease similarities, NCPMDA integrated the known miRNA-disease association network and miRNA family information to restructure the miRNA–miRNA and disease–disease similarity networks is to predict miRNA-disease associations. LOOCV and case studies have shown that integrated information on known association networks and miRNA families aids in the improvement of the predictive performance of NCPDMA. Compared with the current state-of-the-art computational methods for predicting miRNA-disease associations, NCPMDA does not require the use of negative samples. NCPDMA is also a universal method that can be used in simultaneously restructuring the potential associations for all diseases. Specifically, NCPMDA is a non-parametric method and demonstrates an obvious advantage when the known and experimentally verified miRNA-disease associations are very few. Furthermore, NCPMDA can predict related miRNAs of isolated diseases. Therefore, NCPMDA can be a useful resource for the prediction of miRNA-disease associations.

Despite the favorable results obtained using NCPMDA, this study presents some limitations. First, we simply used the similarity of known associations and miRNA family information as variables to restructure the miRNA–miRNA and disease–disease similarity networks; a more reasonable measurement and integration method can improve the performance of NCPMDA. Second, the final prediction score of NCPMDA was computed using the miRNA space projection and disease space projection; obtaining a single measurement or a more accurate integration of results from two different spaces should be prioritized in future studies. Finally, the increase in the number of miRNA-disease associations being experimentally verified advances the development of computational approaches to predict miRNA-disease associations.

## Methods

### Dataset and preprocessing

Two datasets are used in this study. For ease of description, the two datasets are called benchmark dataset and predictive dataset. Each dataset contains four kinds of data, namely, miRNA functional similarities, miRNA family information, disease semantic similarities, and known miRNA-disease associations.

The disease similarities were obtained from the literature^32^. A strict system for disease classification from the MeSH database (http://www.ncbi.nlm.nih.gov/) was obtained, and diseases were described in a DAG, in which the nodes represent the disease itself and its precursor diseases, whereas the link from a parent node to a child node represents the relationship between these nodes. The disease similarity was calculated using disease DAGs, with the assumption that two diseases sharing larger parts of DAGs are more similar. To represent the variables, we used the matrix *DD* to denote disease similarities, where *DD(i, j*) in row *i* and column *j* represents the similarity between diseases *i* and *j*.

The miRNA functional similarity scores were obtained from a reliable webpage (http://www.cuilab.cn/). On the basis of the assumption implying that miRNAs with similar functions tend to be associated with similar diseases, the similarity score of miRNA pairs were measured the similarity of their associated disease DAG (the literature^37^ provides a detailed description of the score calculation). We used a matrix *MM* which denotes the miRNA–miRNA functional similarity, where the variable *MM(i, j*) in row *i* and column *j* is the functional similarity between miRNA *i* and *j*.

The information on miRNA families was obtained from the miRBase^49^ Sequence Database, Release 21 (http://www.mirbase.org/). We used the matrix *FAM* to represent the information on miRNA families, where the variable *FAM(i, j*) in row *i* and column *j* is 1 if miRNA *i* and *j* belong to the same family; otherwise, the value is 0.

In the benchmark dataset, the known miRNA-disease associations were obtained from literature^20^. These associations were collected from the HMDD^12^ and mir2disease^13^ databases. The databases include 271 high-qualityand experimentally verified associations of miRNA deregulation with disease development. However, 19 miRNAs were not found in literature^37^, which left 242 high-quality miRNA-disease associations, including 99 miRNAs and 51 diseases. In the predictive dataset, the known miRNA-disease associations were also acquired^37^ to evaluate the prediction accuracy. The association data included 1616 distinct, high-quality, and experimentally verified human miRNA-disease associations, which were obtained from HMDD in September 2009. The names of different mature miRNAs and diseases were unified, and the records of different miRNA copies were consolidated. Finally, 1395 miRNA-disease associations, including 271 miRNAs and 137 diseases, were obtained. We used the information on the predictive dataset for the evaluation of prediction accuracy and the prediction of new miRNA-disease associations. We preferred to use the updated version of HMDD and other databases (mir2disease and dbDEMC) to verify our prediction. Hence, we did not include the latest association dataset in HMDD and other new associations in other databases. To represent the variables, we used the matrix *AS* signifies the adjacency matrix of miRNA-disease association, where *AS(i, j*) in row *i* and column *j* is 1 if miRNA *i* is associated with disease *j*; otherwise, the value is 0.

### Constructing a miRNA–miRNA similarity network

To compute the relationship between miRNA pairs more efficiently, we integrated the miRNA functional similarities, miRNA family information, and miRNA similarities of known miRNA-disease associations to construct a miRNA–miRNA similarity network.

On the basis of the human miRNA-disease associations (matrix *AS*) considered and the assumption that two miRNAs are associated more with common diseases, and the function of the miRNA pair are more similar, the miRNA similarity of the known associations is calculated using the Jaccard similarity measure, as shown in Equation (1):


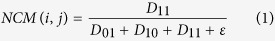


Matrix *NCM* represents the miRNA–miRNA functional similarity scores of known associations, where *NCM(i, j*) indicates the similarity between miRNA *i* and *j*. Where *D*_*11*_ is the total number of variables with a value of 1 in both miRNA *i* and *j*, that is, the total number of diseases simultaneously associated with miRNA *i* and *j*. Similarly, *D*_*01*_ is the total number of variables with a value of 0 in miRNA *i* and 1 in miRNA *j*, whereas *D*_*10*_ is the total number of variables with a value of 1 in miRNA *i* and 0 in miRNA *j*. This study focuses on known miRNA-disease associations; thus, the total number of variables with 0 value in both miRNA *i* and *j* is disregarded. For certain diseases, the number of related-miRNA is only one, and remove this association for LOOCV will lead to *D*_*11*_, *D*_*10*_ and *D*_*01*_ are 0. In Equation (1), ε represents a very small positive real number which does not affect the final score. Its function is to avoid having 0 as the denominator. In our experiments, *ε* is set to 10^−30^. For similar reasons, the use of ε in the back of the article uses the same settings.

By using the miRNA functional similarities (matrix *MM*), miRNA family information (matrix *FAM*), and miRNA similarities of known miRNA-disease associations (matrix *NCM*) as variables, we incorporate them into Equation (2) to construct a miRNA–miRNA similarity network:


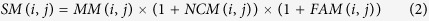


where *SM(i, j*) is the final similarity score of miRNA *i* and *j*. Obviously, if two miRNAs in the known association network are more similar and belong to the same family, the computed score is higher.

### Constructing disease–disease similarity network

Disease semantic similarities and disease similarities of known miRNA-disease associations were applied to construct a disease–disease similarity network.

Parallel to the miRNA similarities of the known miRNA-disease associations and based on the assumption that two diseases associated with more common miRNAs are more similar, we use the Jaccard similarity measure to calculate the disease similarities of known miRNA-disease associations, as shown in Equation (3):


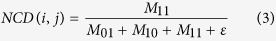


where *M*_*11*_ is the total number of variables with a value of 1 in both diseases *i* and *j*, that is, *M*_*11*_ is the total number of miRNAs simultaneously associated with disease *i* and *j*. Similarly, *M*_*01*_ is the total number of variables with a value of 0 in disease *i* and 1 in disease *j*, whereas *M*_*10*_ is the total number of variables with a value of 1 in disease *i* and 0 in disease *j*. The significance of *ε* is the same as that in the previous equation.

Analogous to Equation (2), we use the values for the disease semantic similarities (matrix *DD*) and the disease similarities of known associations and incorporate them into Equation (4) to construct a disease–disease similarity network:


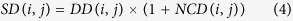


where *SD(i, j*) is the final similarity score of disease *i* and *j*. Evidently, if two diseases in the known association network are more similar, the computed score for them is higher.

### Network Consistency Projection of miRNA-Disease Association (NCPMDA)

**Figure 4:**
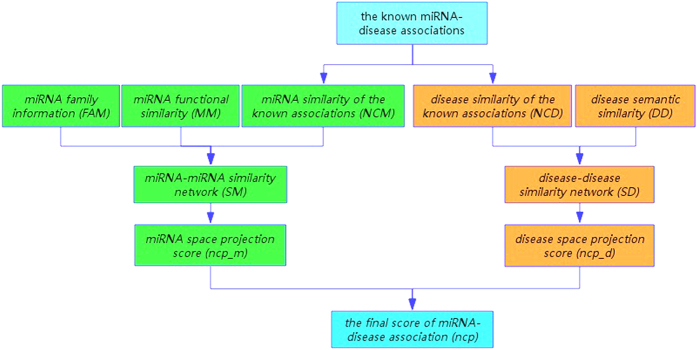
The flowchart of NCPMDA method.

In accordance with the assumption that diseases associated with highly related miRNAs are more similar (and vice versa) and that miRNAs associated with highly related diseases are more similar (and vice versa), we developed the Network Consistency Projection for miRNA-Disease Associations (NCPMDA) method to predict potential miRNA-disease associations. The flowchart of NCPMDA method shows in Fig. 4.

NCPMDA calculates the potential miRNA-disease association score consisting of two network consistency projection scores, miRNA space projection score and disease space projection score, separately. The network consistency mentioned here refers to the higher the spatial similarity of miRNA *i* associated miRNAs in miRNA-miRNA similarity network and disease *j* associated miRNAs in the known miRNA-disease network, the greater the association of miRNA *i* with disease *j*. We use vector space projection to represent it and named miRNA space projection. Similarly, disease space projection measure the association of miRNA *i* and disease *j* in disease space. Considering the miRNA-disease associations not verified by experiment cannot confirm that there is no association and to avoid having 0 as the denominator, we replace 0 in matrix *AS* to *ε*. In our experiments, *ε* is set to 10^−30^. Simultaneously, we use *|C|* to represent the length of vector *C* (the norm of vector *C*). The miRNA space projection score is calculated as


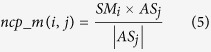


where *SM*_*i*,_ is the *ith* row of matrix *SM* and a vector consisting of the similarities between miRNA *i* and all other miRNAs. Similarly, *AS*_*j*_ is the *jth* column of matrix *AS* and the vector consisting of the associations of disease *j* and all miRNAs. Matrix *ncp_m* is the network consistency projection score of the miRNA similarity network *SM* on the known miRNA-disease association network, *AS*; the variable *ncp_m(i, j*) in row *i* and column *j* is the network consistency projection of *SM*_*i*_ on *AS*_*j*_. Notably, the smaller angle between *SM*_*i*_ and *AS*_*j*_, the more miRNAs associated with disease *j*, and the more similar miRNAs and miRNA *i* are, the greater the network consistency projection score *ncp_m(i, j*) is.

Similarly, the disease space projection score is calculated as follows:


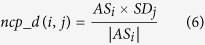


where *SD*_*j*_ is the *jth* column of matrix *SD*, the vector comprising the similarities of disease *j* and all other diseases. Similarly, *AS*_*i*_ is the *ith* row of matrix *AS*, which consists of the associations of miRNA *i* and all diseases. Matrix *ncp_d* is the projection of the disease similarity network, *SD*, on the known miRNA-disease association network, *AS; ncp_d(i, j*) in row *i* and column *j* is the network consistency projection of *SD*_*j*_ on *AS*_*i*_. Remarkably, the smaller angle between *SD*_*j*_ and *AS*_*i*_, the more diseases are associated with miRNA *i*, and the more similar these diseases and disease *j* are, the greater the network consistency projection score *ncp_d(i, j*) is.

Finally, the miRNA space projection score and disease space projection score are combined and normalized as shown below:


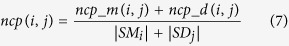


where *ncp(i, j*) is the final score of network consistency projection of miRNA *i* and disease *j; ncp_m(i, j*) and *ncp_d(i, j*) are the miRNA space projection score and disease space projection score of miRNA *i* and disease *j*, respectively. The final score is used to predict miRNA-disease association. If only NCPMDA in miRNA space projection (remove *ncp_d(i, j*) and *|SD*_*j*_|) is considered, the final score of miRNA *i* and disease *j* is the cosine similarity of space vector *SM*_*i*_ and *AS*_*j*._ Similarly, the cosine similarity of space vector *SD*_*j*_ and *AS*_*i*_ is the final score of miRNA *i* and disease *j* when considering NCPMDA in disease space projection only.

## Additional Information

**How to cite this article**: Gu, C. *et al*. Network Consistency Projection for Human miRNA-Disease Associations Inference. *Sci. Rep.* **6**, 36054; doi: 10.1038/srep36054 (2016).

## Supplementary Material

Supplementary Information

## Figures and Tables

**Table 1:** The top 40 potential miRNA-disease associations predicted by NCPMDA and the confirmation by HMDD, mir2disease and dbDEMC are listed here.

Rank	MiRNA	Disease	Evidences
1	hsa-mir-34a	Ovarian Neoplasms	HMDD
2	hsa-mir-223	Breast Neoplasms	HMDD, dbDEMC
3	hsa-let-7e	Breast Neoplasms	HMDD, dbDEMC
4	hsa-mir-127	Lung Neoplasms	HMDD, dbDEMC
5	hsa-mir-127	Melanoma	mir2disease
6	hsa-let-7b	Breast Neoplasms	HMDD, dbDEMC
7	hsa-mir-141	Melanoma	HMDD, mir2disease
8	hsa-mir-16	Breast Neoplasms	HMDD, dbDEMC
9	hsa-let-7d	Ovarian Neoplasms	HMDD, mir2disease
10	hsa-mir-221	Neoplasms	HMDD, dbDEMC
11	hsa-mir-18a	Prostatic Neoplasms	Unconfirmed
12	hsa-mir-155	Melanoma	HMDD
13	hsa-mir-126	Breast Neoplasms	HMDD, mir2disease, dbDEMC
14	hsa-let-7i	Breast Neoplasms	HMDD, mir2disease, dbDEMC
15	hsa-mir-21	Melanoma	HMDD
16	hsa-let-7f	Ovarian Neoplasms	HMDD, mir2disease
17	hsa-mir-222	Ovarian Neoplasms	mir2disease
18	hsa-mir-145	Melanoma	HMDD
19	hsa-mir-18a	Pancreatic Neoplasms	HMDD
20	hsa-mir-92b	Breast Neoplasms	dbDEMC
21	hsa-mir-191	Breast Neoplasms	HMDD, mir2disease, dbDEMC
22	hsa-mir-101	Breast Neoplasms	HMDD, mir2disease, dbDEMC
23	hsa-mir-221	Lung Neoplasms	HMDD, dbDEMC
24	hsa-mir-223	Neoplasms	dbDEMC
25	hsa-mir-221	Ovarian Neoplasms	HMDD
26	hsa-mir-30c	Melanoma	Unconfirmed
27	hsa-mir-145	Pancreatic Neoplasms	HMDD
28	hsa-mir-222	Neoplasms	HMDD, dbDEMC
29	hsa-mir-92a	Breast Neoplasms	HMDD
30	hsa-mir-222	Colonic Neoplasms	dbDEMC
31	hsa-mir-143	Ovarian Neoplasms	Unconfirmed
32	hsa-mir-106a	Neoplasms	HMDD, dbDEMC
33	hsa-mir-25	Lung Neoplasms	HMDD, dbDEMC
34	hsa-mir-125b	Neoplasms	HMDD,dbDEMC
35	hsa-mir-199a	Colonic Neoplasms	Unconfirmed
36	hsa-mir-200b	Lung Neoplasms	HMDD, mir2disease, dbDEMC
37	hsa-let-7c	Breast Neoplasms	HMDD, dbDEMC
38	hsa-let-7c	Ovarian Neoplasms	HMDD,mir2disease
39	hsa-mir-155	Carcinoma, Hepatocellular	HMDD,dbDEMC
40	hsa-mir-125a	Melanoma	HMDD, mir2disease

Thirty-six of the top 40 associations have been confirmed by various databases.
