# Supplementary material for: Network Consistency Projection for Human miRNA-Disease Associations Inference
Source: Sci Rep. 2016 Oct 25;6:36054. doi: 10.1038/srep36054 (PMC5078764; doi:10.1038/srep36054)
Supplement: Supplementary Information [file srep36054-s1.pdf]

Network Consistency Projection for Human miRNA-Disease Associations Inference

Changlong Gu[1], Bo Liao[1,*], Xiaoying Li[1], and Keqin Li[2]

[1]College of Information Science and Engineering, Hunan University, Changsha, Hunan 410082, China

[2]Department of Computer Science, State University of New York, New Paltz, New York 12561, USA

*Corresponding authors

Email: dragonbw@163.com

Supplementary Information

S1. The top 40 potential breast cancer-related miRNAs predicted by NCPMDA and the confirmation of these associations. Thirty-eight of the top 40 breast cancer-related miRNAs have been confirmed based on the update HMDD, mir2disease and dbDEMC databases.

rank	miRNA	evidences
1	hsa-mir-223	HMDD,dbDEMC
2	hsa-let-7e	HMDD,dbDEMC
3	hsa-let-7b	HMDD,dbDEMC
4	hsa-mir-16	HMDD,dbDEMC
5	hsa-mir-126	HMDD,mir2disease,dbDEMC
6	hsa-let-7i	HMDD,mir2disease,dbDEMC

7	hsa-mir-92b	dbDEMC
8	hsa-mir-191	HMDD,mir2disease,dbDEMC
9	hsa-mir-101	HMDD,mir2disease,dbDEMC
10	hsa-mir-92a	HMDD
11	hsa-let-7c	HMDD,dbDEMC
12	hsa-let-7g	HMDD,dbDEMC
13	hsa-mir-18b	HMDD,dbDEMC
14	hsa-mir-106a	dbDEMC
15	hsa-mir-373	HMDD,mir2disease,dbDEMC
16	hsa-mir-203	HMDD,mir2disease,dbDEMC
17	hsa-mir-181a	HMDD,mir2disease,dbDEMC
18	hsa-mir-29c	HMDD,mir2disease,dbDEMC
19	hsa-mir-24	HMDD,dbDEMC
20	hsa-mir-30e	Unconfirmed
21	hsa-mir-32	dbDEMC
22	hsa-mir-130a	dbDEMC
23	hsa-mir-99b	dbDEMC
24	hsa-mir-15b	dbDEMC
25	hsa-mir-372	dbDEMC
26	hsa-mir-128b	mir2disease
27	hsa-mir-182	HMDD,mir2disease,dbDEMC
28	hsa-mir-100	HMDD,dbDEMC

29	hsa-mir-199b	HMDD,dbDEMC
30	hsa-mir-150	dbDEMC
31	hsa-mir-335	HMDD,mir2disease,dbDEMC
32	hsa-mir-192	dbDEMC
33	hsa-mir-142	Unconfirmed
34	hsa-mir-98	mir2disease,dbDEMC
35	hsa-mir-195	HMDD,mir2disease,dbDEMC
36	hsa-mir-224	HMDD,dbDEMC
37	hsa-mir-30a	HMDD,mir2disease
38	hsa-mir-27a	HMDD,mir2disease,dbDEMC
39	hsa-mir-23b	HMDD,dbDEMC
40	hsa-mir-95	dbDEMC

S2. The top 40 potential HCC related miRNAs predicted by NCPMDA and the confirmation of these associations. Thirty-seven of the top 40 breast cancer-related miRNAs have been confirmed based on the update HMDD, mir2disease and dbDEMC databases.

rank	miRNA	evidences
1	hsa-mir-155	HMDD,mir2disease,dbDEMC
2	hsa-mir-141	HMDD,mir2disease
3	hsa-mir-143	mir2disease,dbDEMC

4	hsa-mir-126	HMDD,mir2disease,dbDEMC
5	hsa-let-7a	HMDD,mir2disease,dbDEMC
6	hsa-mir-16	HMDD,mir2disease,dbDEMC
7	hsa-mir-181b	HMDD,mir2disease
8	hsa-mir-191	HMDD,dbDEMC
9	hsa-mir-106b	HMDD,mir2disease,dbDEMC
10	hsa-mir-25	HMDD,mir2disease,dbDEMC
11	hsa-mir-9	mir2disease
12	hsa-mir-15a	HMDD,mir2disease,dbDEMC
13	hsa-mir-93	HMDD,mir2disease,dbDEMC
14	hsa-mir-146b	HMDD
15	hsa-mir-194	mir2disease
16	hsa-let-7i	HMDD,dbDEMC
17	hsa-mir-34c	HMDD
18	hsa-mir-214	HMDD,mir2disease,dbDEMC
19	hsa-mir-29b	HMDD
20	hsa-mir-132	mir2disease
21	hsa-let-7b	HMDD,mir2disease
22	hsa-let-7d	HMDD,mir2disease
23	hsa-mir-30c	HMDD,mir2disease
24	hsa-mir-429	Unconfirmed
25	hsa-let-7c	HMDD,mir2disease,dbDEMC

26	hsa-let-7g	HMDD,mir2disease
27	hsa-let-7f	HMDD,mir2disease
28	hsa-mir-205	HMDD,mir2disease
29	hsa-mir-135b	Unconfirmed
30	hsa-mir-200c	HMDD
31	hsa-mir-29a	HMDD,dbDEMC
32	hsa-mir-34b	Unconfirmed
33	hsa-mir-15b	HMDD,dbDEMC
34	hsa-mir-29c	HMDD,dbDEMC
35	hsa-mir-30d	HMDD,dbDEMC
36	hsa-mir-128b	mir2disease
37	hsa-mir-24	HMDD,mir2disease
38	hsa-mir-196a	HMDD
39	hsa-mir-151	mir2disease
40	hsa-mir-210	HMDD,dbDEMC

S3. The top 40 breast cancer-related miRNAs predicted by NCPMDA with removed all known breast cancer-miRNA associations and the confirmation of these associations. Thirty-seven of the top 40 breast cancer-related miRNAs have been confirmed based on the update HMDD, mir2disease and dbDEMC databases.

rank	miRNA	evidences
1	hsa-let-7e	HMDD,dbDEMC
2	hsa-let-7b	HMDD,dbDEMC
3	hsa-mir-16	HMDD,dbDEMC
4	hsa-let-7i	HMDD,mir2disease,dbDEMC
5	hsa-mir-223	HMDD,dbDEMC
6	hsa-let-7c	HMDD,dbDEMC
7	hsa-let-7g	HMDD,dbDEMC
8	hsa-mir-92a	HMDD
9	hsa-mir-101	HMDD,mir2disease,dbDEMC
10	hsa-mir-191	HMDD,mir2disease,dbDEMC
11	hsa-mir-203	HMDD,mir2disease,dbDEMC
12	hsa-mir-106a	dbDEMC
13	hsa-mir-92b	dbDEMC
14	hsa-mir-18b	HMDD,dbDEMC
15	hsa-mir-126	HMDD,mir2disease,dbDEMC
16	hsa-mir-29c	HMDD,mir2disease,dbDEMC
17	hsa-mir-15b	dbDEMC
18	hsa-mir-181a	HMDD,mir2disease,dbDEMC
19	hsa-mir-128b	mir2disease
20	hsa-mir-373	HMDD,mir2disease,dbDEMC
21	hsa-mir-24	HMDD,dbDEMC

22	hsa-mir-224	HMDD,dbDEMC
23	hsa-mir-142	Unconfirmed
24	hsa-mir-32	dbDEMC
25	hsa-mir-150	dbDEMC
26	hsa-mir-449b	Unconfirmed
27	hsa-mir-449a	Unconfirmed
28	hsa-mir-137	HMDD,dbDEMC
29	hsa-mir-99b	dbDEMC
30	hsa-mir-192	dbDEMC
31	hsa-mir-195	HMDD,mir2disease,dbDEMC
32	hsa-mir-124	HMDD,dbDEMC
33	hsa-mir-376a	dbDEMC
34	hsa-mir-301b	HMDD
35	hsa-mir-144	dbDEMC
36	hsa-mir-301a	HMDD
37	hsa-mir-23b	HMDD,dbDEMC
38	hsa-mir-31	HMDD,mir2disease,dbDEMC
39	hsa-mir-183	HMDD,dbDEMC
40	hsa-mir-135a	HMDD,dbDEMC

S4. The top 40 HCC-related miRNAs predicted by NCPMDA with removed all known HCC-miRNA associations and the confirmation of these associations. Thirty-six of the top 40 HCC-related miRNAs have been confirmed based on the update HMDD, mir2disease and dbDEMC databases.

rank	miRNA	evidences
1	hsa-let-7a	HMDD,mir2disease,dbDEMC
2	hsa-mir-155	HMDD,mir2disease,dbDEMC
3	hsa-let-7d	HMDD,mir2disease
4	hsa-mir-181b	HMDD,mir2disease
5	hsa-let-7b	HMDD,mir2disease
6	hsa-mir-15a	HMDD,mir2disease,dbDEMC
7	hsa-mir-16	HMDD,mir2disease,dbDEMC
8	hsa-let-7i	HMDD,dbDEMC
9	hsa-let-7f	HMDD,mir2disease
10	hsa-mir-141	HMDD,mir2disease
11	hsa-let-7c	HMDD,mir2disease,dbDEMC
12	hsa-mir-143	mir2disease,dbDEMC
13	hsa-let-7g	HMDD,mir2disease
14	hsa-mir-30c	HMDD,mir2disease
15	hsa-mir-15b	HMDD,dbDEMC

16	hsa-mir-25	HMDD,mir2disease,dbDEMC
17	hsa-mir-194	mir2disease
18	hsa-mir-9	mir2disease
19	hsa-mir-191	HMDD,dbDEMC
20	hsa-mir-205	HMDD,mir2disease
21	hsa-mir-181a	HMDD,mir2disease,dbDEMC
22	hsa-mir-126	HMDD,mir2disease,dbDEMC
23	hsa-mir-132	mir2disease
24	hsa-mir-196a	HMDD
25	hsa-mir-135b	Unconfirmed
26	hsa-mir-146b	HMDD
27	hsa-mir-200c	HMDD
28	hsa-mir-29b	HMDD
29	hsa-mir-429	Unconfirmed
30	hsa-mir-214	HMDD,mir2disease,dbDEMC
31	hsa-mir-106b	HMDD,mir2disease,dbDEMC
32	hsa-mir-34c	HMDD
33	hsa-mir-93	HMDD,mir2disease,dbDEMC
34	hsa-mir-302b	HMDD
35	hsa-mir-128b	mir2disease
36	hsa-mir-34b	Unconfirmed
37	hsa-mir-449b	HMDD

38	hsa-mir-449a	Unconfirmed
39	hsa-mir-29c	HMDD,dbDEMC
40	hsa-mir-137	mir2disease
